# Cell‐free DNA kinetics in response to muscle‐damaging exercise: A drop jump study

**DOI:** 10.1113/EP091986

**Published:** 2024-06-14

**Authors:** Ema Juškevičiūtė, Elmo Neuberger, Nerijus Eimantas, Kirsten Heinkel, Perikles Simon, Marius Brazaitis

**Affiliations:** ^1^ Institute of Sport Science and Innovations Lithuanian Sports University Kaunas Lithuania; ^2^ Department of Sports Medicine, Disease Prevention and Rehabilitation Johannes Gutenberg University Mainz Mainz Germany

**Keywords:** blood markers, cell‐free DNA, eccentric exercise, muscle damage

## Abstract

A significant increase in circulating cell‐free DNA (cfDNA) occurs with physical exercise, which depends on the type of exertion and the duration. The aims of this study were as follows: (1) to investigate the time course of cfDNA and conventional markers of muscle damage from immediately after to 96 h after muscle‐damaging exercise; and (2) to investigate the relationship between cfDNA and indicators of primary (low‐frequency fatigue and maximal voluntary isometric contraction) and secondary (creatine kinase and delayed‐onset muscle soreness) muscle damage in young healthy males. Fourteen participants (age, 22 ± 2 years; weight, 84.4 ± 11.2 kg; height, 184.0 ± 7.4 cm) performed 50 intermittent drop jumps at 20 s intervals. We measured cfDNA and creatine kinase concentrations, maximal voluntary isometric contraction torque, low‐frequency fatigue and delayed‐onset muscle soreness before and at several time points up to 96 h after exercise. Plasma cfDNA levels increased from immediately postexercise until 72 h postexercise (*P* < 0.01). Elevation of postexercise cfDNA was correlated with both more pronounced low‐frequency fatigue (*r* = −0.52, *P* = 3.4 × 10^−11^) and delayed‐onset muscle soreness (*r* = 0.32, *P* = 0.00019). Levels of cfDNA change in response to severe primary and secondary muscle damage after exercise. Levels of cfDNA exhibit a stronger correlation with variables related to primary muscle damage than to secondary muscle damage, suggesting that cfDNA is a more sensitive marker of acute loss of muscle function than of secondary inflammation or damaged muscle fibres.

## Abstract (highlights)

- **What is the central question of this study?**
  How do plasma cell‐free DNA (cfDNA) levels change in response to primary and secondary muscle damage when muscle‐damaging exercise is performed without metabolic stress?
- **What is the main finding and its importance?**
  In this study, we found that cfDNA concentration increases following severe primary and secondary muscle damage after 50 drop jumps. However, acute postexercise increases in cfDNA were higher than following secondary muscle damage and were more correlated with variables related to primary muscle damage (low‐frequency fatigue). This suggests that muscle‐damaging exercise causing no accumulation of metabolites can lead to acute and delayed increases in cfDNA levels. However, they are more sensitive to primary muscle damage.

## 1 INTRODUCTION

Performing unaccustomed exercise can lead to muscle damage, especially when muscles perform eccentric contractions (Chalchat et al., 2022). In contrast to concentric or isometric contractions, eccentric lengthening contractions cause more profound sarcomere damage that leads to a more severe inflammatory response (Markus et al., 2021). Drop jumps (DJs) are a very common plyometric exercise used in sports training that are eccentrically biased. They are also one of the most powerful tools for the examination of exercise‐induced muscle damage (Dias et al., 2022; Tanabe et al., 2022). Mechanically demanding contractions, particularly those in muscles unaccustomed to eccentric contractions (Dargeviciute et al., 2013; Newham et al., 1987), such as those used in DJs, can result in disruption of muscle‐fibre structure, protein leakage, prolonged force depression, delayed‐onset muscle soreness (DOMS) and inflammation (Clarkson & Hubal, 2002; Peake et al., 2017). Several markers describe these changes in sports science; however, the mechanisms by which they function or are released are highly distinct.

Increases in plasma biomarkers, such as creatine kinase (CK), C‐reactive protein or myoglobin, are often used to evaluate the magnitude of exercise‐induced muscle damage (Dias et al., 2022; Dupuy et al., 2018). However, there has been much debate in the last few years about the use of CK, e.g., owing to its slow reaction time and declining response to increased training levels, which, in turn, decrease its sensitivity to changes in training load (Haller et al., 2023; Saw et al., 2016). Previous studies have suggested reduced muscle force after eccentric exercise as the most appropriate indirect marker (Damas et al., 2016; Paulsen et al., 2012). However, the relationship between levels of circulating muscle‐specific proteins (myoglobin and CK) and muscle function appears to be inconsistent (Friden & Lieber, 2001). As an example, loss of force and CK activity have different time courses. Muanjai et al. (2019) reported that a decrease in force and a rise in CK levels represent different types of muscle damage, termed primary and secondary, respectively. Low‐frequency fatigue (LFF; the ratio of forces generated at 20 and 100 Hz stimulation), which is attributable to decreased sarcoplasmic reticulum Ca^2+^ release and/or diminished myofibrillar Ca^2+^ sensitivity, in addition to an increase in reactive oxygen species, can also be used as an indirect marker of primary damage (Wyckelsma et al., 2020). Secondary damage results in structural changes to the muscle fibre. Furthermore, damage might be attributable to white blood cell infiltration (mainly neutrophil granulocytes) of the muscle or increased intracellular Ca^2+^ activating phospholipase and protease enzymes (Yamada et al., 2018). Neither of the discussed markers (CK or LFF) is sensitive enough to respond to both acute exercise and secondary muscle damage, making alternative biomarkers necessary.

Cell‐free DNA (cfDNA), which is DNA released from cells that circulate in the bloodstream, could potentially serve as a substitute marker for exercise‐induced muscle damage (Andreatta et al., 2018). Cell‐free DNA is known to be a pro‐inflammatory load‐dependent marker of aerobic and anaerobic exercise (Breitbach et al., 2012; Huminska‐Lisowska et al., 2021). An increase in the level of this marker is associated with the activation and recruitment of immune cells (Korabecna et al., 2020). In healthy individuals, exercise is the primary external variable contributing to increased cfDNA levels (Andreatta et al., 2018; Atamaniuk et al., 2010; Haller et al., 2017). Many of the published studies have used protocols that require muscles to perform concentric and eccentric contractions, such as cycling (Tug et al., 2017), aerobic running (Haller et al., 2017), marathon (Atamaniuk et al., 2008) or lifting exercise (Atamaniuk et al., 2010), and light and heavy resistance training (Andreatta et al., 2018), meaning that simultaneous metabolic stress (accumulation of metabolites, i.e., lactate, H^+^ ions in muscle cells and altered calcium homeostasis) and mechanical stress (structural changes involving sarcomere, cytoskeletal and membrane damage; disruption of excitation–contraction coupling) would occur. Only one study focused solely on eccentric training, where high‐intensity and low‐intensity eccentric cycling exercise was compared (Mavropalias et al., 2021). Although the protocol involved eccentric exercise, the participants performed high‐intensity cycling, in which the accumulation of metabolites would have been present but lower than that of exercise in most previous studies (Juškevičiūtė et al., 2023; Tug et al., 2017). Hence, the extent to which the increase in cfDNA is caused by mechanically demanding exercise alone is unclear. To our knowledge, no studies have investigated the effects of DJs on cfDNA concentrations. In the present study, we wanted to observe how mechanically demanding exercise, without placing the organism under metabolic stress, affects cfDNA levels. It was thought that muscle contraction without the accumulation of metabolites, such as lactate, could increase cfDNA levels, first, because of physical exertion and, second, because of neutrophil infiltration into tissues owing to local inflammation and oxidative stress (Mortaz et al., 2018), because neutrophils are essential in the release of cfDNA (Moss et al., 2018).

Here, we aimed to examine the time course of plasma cfDNA levels after intermittent DJs and assess the extent to which cfDNA increases in the absence of metabolic stress. We also analysed whether plasma cfDNA levels change in response to DOMS and levels of CK, and whether there are associations between plasma cfDNA levels and neuromuscular fatigue indices. We aimed to test the hypothesis that cfDNA reacts to acute exercise (primary damage), in addition to delayed muscle damage (secondary damage).

## 2 MATERIALS AND METHODS

### 2.1 Ethical approval

The trial procedures were authorized by the Kaunas Region Biomedical Research Ethics Committee (no. BE‐2‐35) and the Human Ethics Committee Rhineland‐Palatinate (no. 2019‐14256), and all experiments were performed in accordance with the *Declaration of Helsinki*. All participants were informed orally and in writing about the experimental protocol and the aim of the study and gave written agreement to participate.

### 2.2 Participants and familiarization

Healthy male participants were recruited (mean ± SD; age, 22 ± 2 years; weight, 84.4 ± 11.2 kg; height, 184.0 ± 7.4 cm; *n* = 14) who did not participate in any official exercise or sports programme but were physically active by the standard of participating in recreational physical activity two or three times per week. Participants were asked to abstain from any strength and conditioning programme for 72 h before the pretest and during the test week. Exclusion criteria for participation in the study included individuals who currently take any medications or suffer from neuromuscular disease or cardiovascular disease. Three days before the initial study, each participant completed a familiarization session to become familiar with all the measurements. During the same visit, tolerance to electrical stimulation was assessed. As a precaution, participants performed only one or two DJs for learning purposes before the trial to avoid muscle damage.

### 2.3 Drop jumps

Every participant performed 50 DJs at intervals of one every 20 s. The interval of 20 s was chosen to minimize metabolic stress. Fifty DJs were chosen because they induce sufficient primary and secondary damage (Skurvydas et al., 2011).

**FIGURE 1:**
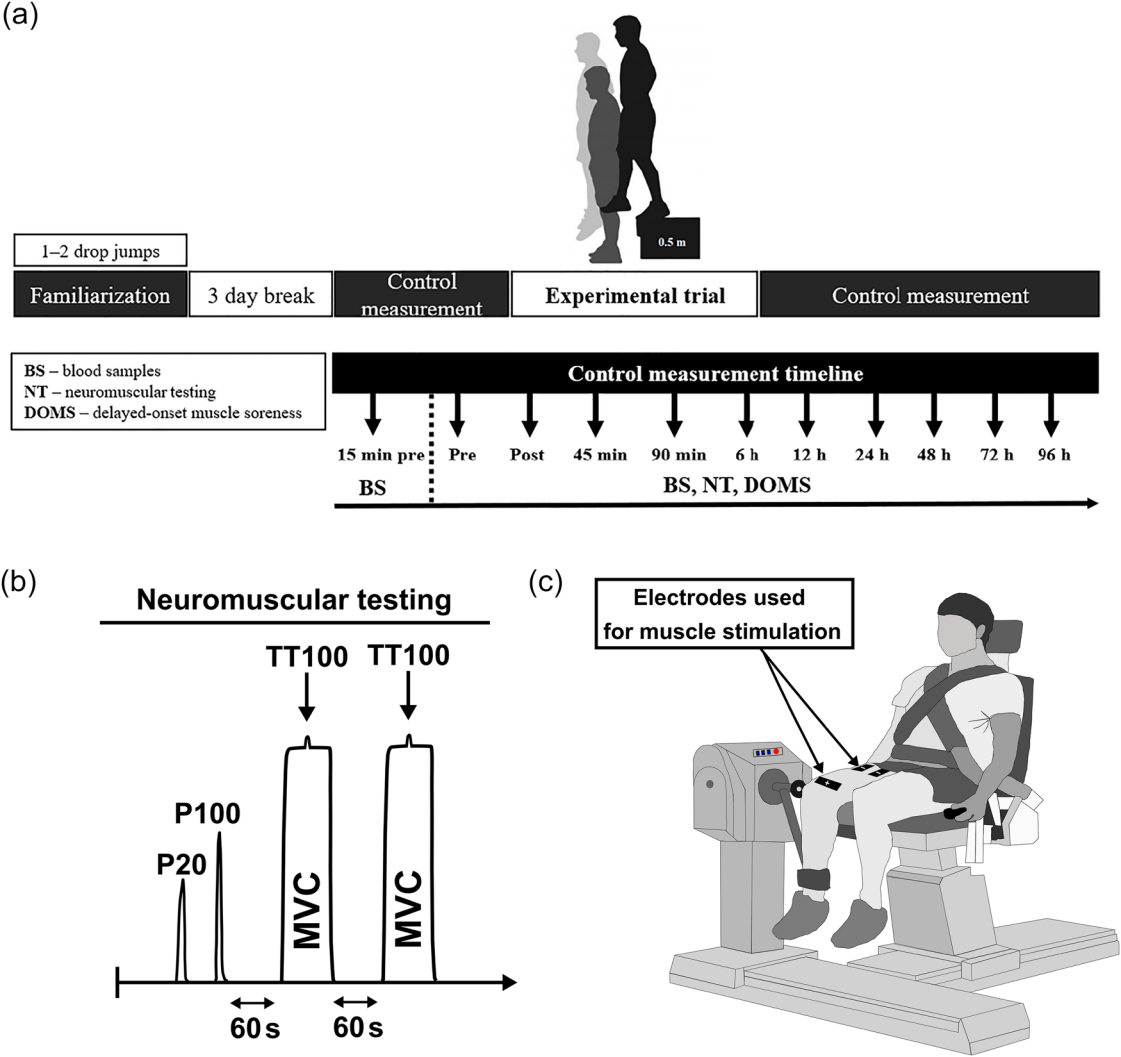
(a) Experimental design. (b) Neuromuscular testing. (c) Position of electrodes placed on the quadriceps. Abbreviations: BS, blood samples; DOMS, delayed‐onset muscle soreness; MVIC, maximal voluntary isometric contraction; NT, neuromuscular testing; P20, electrical stimulation (1 s stimulation) at 20 Hz; P100, electrical stimulation (1 s stimulation) at 100 Hz; TT, 250 ms test train stimulation at 100 Hz.

Each subject underwent exercise testing between 08.00 and 10.00 h. Upon arrival at the laboratory, subjects were asked to sit calmly for 10 min in the room. Blood samples were taken, then subjects performed an 8−10 min warm‐up on an electrically braked cycle ergometer (Ergo‐Fit, Pirmasens, Germany) with a pedalling frequency that ranged between 60 and 70 r.p.m. After warming up, the participants underwent neuromuscular tests, and blood samples were collected again (pre‐exercise). After that, the DJs were conducted (they were performed only once, on the first day of the study). Neuromuscular tests and blood collection were repeated immediately after exercise and at +45 min, +90 min, +6 h and +12 h postexercise and on the following days (24, 48, 72 and 96 h postexercise) (Figure 1a). Subjects performed DJs from a height of 0.5 m to a 90^°^ knee angle, with immediate maximal vertical rebound and with 20 s rest between each DJ. During the experiment, an experienced investigator controlled the knee angle visually. Each subject placed their hands on their hips to isolate the contribution from the upper limbs. The subjects were verbally encouraged to jump as high as they could. Participants stepped up to the platform with their left leg, and the force of muscle contraction in the right leg was tested. A contact mat (Newtest Powertimer Testing system, Oulu, Finland) was used for performing the DJs. For the next 4 days, participants underwent neuromuscular tests as described below, and blood samples were taken.

### 2.4 Neuromuscular testing

Neuromuscular testing, consisting of electrically evoked torque and maximal voluntary isometric contraction (MVIC) of the knee extensors, is illustrated in Figure 1b. The participants sat upright on the seat of an isokinetic dynamometer (System 4; Biodex Medical Systems, Shirley, NY, USA) calibrated according to the manufacturer's recommendations. Belts were attached to the shank, trunk and shoulders to stabilize them. The dynamometer was set with the knee joint positioned at an angle of 90° (180° = full extension) during MVIC and electrical stimulations (Treigyte et al., 2024).

Electrical stimulations were applied using three 12 cm × 8 cm carbonized rubber surface electrodes (MARP Electronic), lubricated with electrode gel (EGG‐EEG Gel, Modi'in, Israel). Two electrodes were positioned vertically and transversely across the width of the proximal portion of quadriceps muscles, and the third one covered the distal portion of the quadriceps muscles above the patella (Figure 1c). To ensure similar electrode placement during each measurement, the electrode sites were marked with a permanent marker. In between experiments, participants were instructed not to erase these marks. An electrical stimulator (Digitimer DS7A; Digitimer, Welwyn Garden City, UK) was connected to the electrodes to deliver 0.5 ms square‐wave pulses at a constant current set at 100 mA and constant voltage limit set at 200 V (Eimantas et al., 2022).

Neuromuscular testing began with two electrical stimulations separated by 3 s of rest: 1 s stimulation at 20 Hz (P20) and 1 s stimulation at 100 Hz (P100). Peak torques were determined for these electrical stimulations, and P20/P100 was calculated. Directly after these stimulations, two MVICs of the knee extensor muscles were performed and separated by a rest period of 1 min. The MVIC torque corresponded to the maximum torque after 2−3 s. The participants were verbally encouraged to exert and maintain maximal effort for ∼5 s, and a 250 ms test train stimulation at 100 Hz was superimposed on voluntary contraction 3−4 s into the MVIC. The central activation ratio (CAR), a measure of voluntary activation level, was calculated as (MVIC torque/total peak torque generated with the superimposed 250 ms test train stimulation) × 100 (Treigyte et al., 2024). The highest MVIC torque value from two attempts was used for further analysis.

### 2.5 Muscle soreness

Participants subjectively assessed muscle soreness (DOMS) using a visual scale of 0–10, where 0 represented no pain and 10 indicated intolerably intense pain (Lau et al., 2015). Participants were requested to evaluate the severity of soreness in their exercised quadriceps during two or three squats (Skurvydas et al., 2011). Muscle soreness was assessed pre‐ and postexercise and at +45 min, +90 min, +6 h, +12 h, +24 h, +48 h, +72 h and +96 h after the DJs.

### 2.6 Blood sampling and processing

Fifteen minutes and immediately before exercise, immediately postexercise and at +45 min, +90 min, +6 h, +12 h, +24 h, +48 h, +72 h and +96 h after exercise, a 9 mL sample of venous blood was collected in EDTA‐collection tubes (EDTA Monovettes; Sarstedt, Germany) and were immediately centrifuged at 1600*g* and 4°C for 10 min. Plasma was transferred to a fresh tube, followed by a second 10 min of centrifugation at 16,000*g* and 4°C. Aliquots were stored at −20°C until further analysis.

### 2.7 Plasma creatine kinase and blood lactate

Blood lactate concentration was measured before exercise, immediately after exercise and at 45 min postexercise using an *Accutrend* portable lactate analyser (Roche, Germany). Creatine kinase activity was measured using an automatic biochemical analyser (Spotchem EZ SP‐4430; Arkray, Kyoto, Japan) at the 11 time points described above.

### 2.8 Quantification of cfDNA

Concentrations of venous cfDNA were quantified by analysing unpurified plasma via quantitative real‐time (qPCR). Direct qPCR is based on the amplification of one length of an abundant L1PA2 repeat, which is a subfamily of the human long interspersed element of class 1. L1PA2 sequences are distributed over all chromosomes and constitute almost 17% of the human genome (Beck et al., 2010). In brief, diluted plasma (1:10 in H_2_O) was used as a template for qPCR. The amplification was based on primers targeting a 90 bp fragment (5ʹ‐TGCCGCAATAAACATACGTG‐3ʹ and 5ʹ‐GACCCAGCCATCCCATTAC‐3ʹ) of human long interspersed nuclear elements of the L1PA2 family. Samples were analysed with a CFX384 Touch Real‐Time PCR system (Bio‐Rad, München, Germany) using the following protocol: 2 min incubation at 98°C, followed by 35 cycles of denaturation at 94°C for 10 s, annealing at 64°C for 40 s and extension at 75°C for 10 s (Neuberger et al., 2022). All samples were run in triplicate. If the triplicates of the samples showed an SD of the quantification cycle (*Cq*) > 0.4, native plasma samples were rediluted and reanalysed.

### 2.9 Data analysis

The qPCR data were captured using the CFX Manager Software, v.3.1 (Bio‐Rad, Hercules, CA, USA) and Microsoft Excel, 2016. Statistical analysis was performed using R software, v.4.0.3. Data are presented as the mean ± SD. The assumption of normality was assessed using the Shapiro–Wilk normality test. A one‐way repeated‐measures ANOVA was used to assess differences between time points. A significant test was followed by *post hoc* tests or Wilcoxon rank‐sum tests, for normally and non‐normally distributed data, respectively. A Bonferroni–Holm adjustment method was used for *P*‐value correction. Correlations between normally distributed and non‐normally distributed data were investigated using Pearson and Spearman correlation tests. The ggplot2 package v.3.2.2 was used for graphical illustrations. We considered *P*‐values < 0.05 to be statistically significant.

## 3 RESULTS

All 14 participants accomplished 50 DJs. Neuromuscular testing was completed, and blood samples and subjective reports of muscle soreness were collected before and after exercise and for the ensuing 4 days.

The height of DJs from the first DJ to the last DJ was not significantly different (30.37 ± 5.55 and 36.39 ± 6.83 cm, respectively, *P* > 0.05).

### 3.1 Voluntary and electrically evoked muscle torque

**FIGURE 2:**
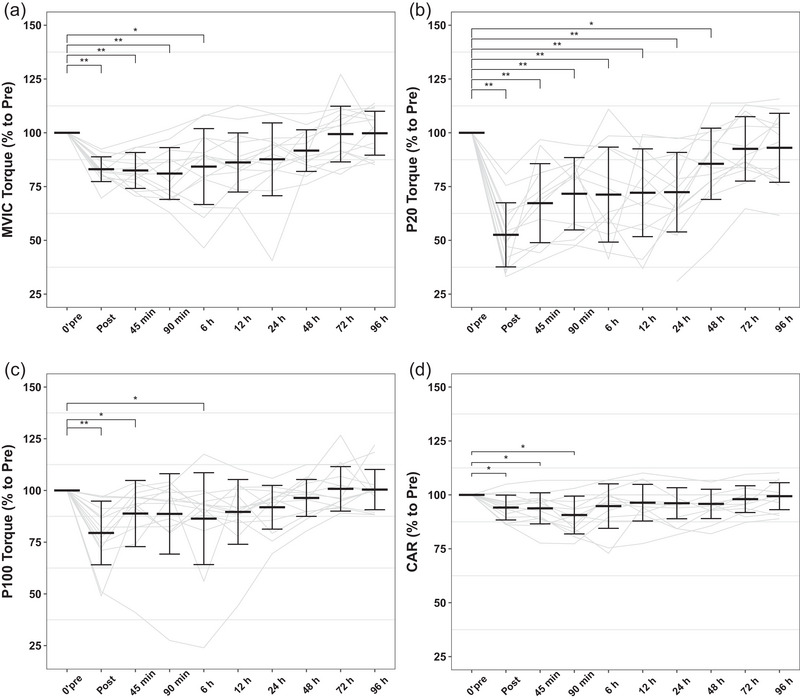
(a) MVC torque. (b) P20 torque. (c) P100 torque. (d) CAR. Data are expressed relative to baseline values, which were set to 100% in each experiment. Data are shown as means ± SD and individual values, *n* = 14. *P < 0.05, **P < 0.01: differences from the Pre time point. Abbreviations: CAR, central activation ratio; MVIC, maximal voluntary isometric contraction; P20, 1 s stimulation at 20 Hz; P100, 1 s stimulation at 100 Hz.

Data for P20, P100, MVIC torques, CAR and LFF were expressed relative to baseline values set to 100% in each experiment. The within one‐way repeated‐measures ANOVA showed that the effects of time (*P *< 0.01) on P20, P100 and MVIC were significant (Figure 2). The MVIC torques were decreased to 83.1% ± 5.75% directly after exercise (*P *< 0.001) and were progressively restored to the baseline values over the recovery period (time effect: *P *< 0.001; Figure 2a). There was a slight decrease in CAR immediately after exercise (94.1% ± 5.76%, *P *< 0.05), 45 min postexercise (93.8% ± 7.21%, *P *< 0.05) and 90 min postexercise (90.7% ± 8.77%, *P *< 0.05), but it was restored after 6 h (*P *> 0.05) (Figure 2d).

**FIGURE 3:**
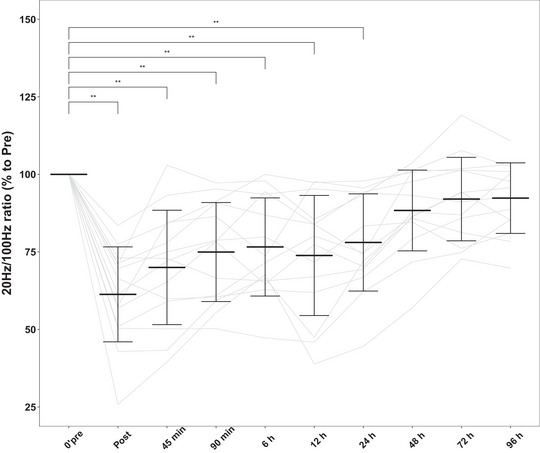
Low‐frequency fatigue (ratio of 20 Hz to 100 Hz electrically stimulated torques). Data are expressed relative to baseline values, which were set to 100% in each experiment. Data are shown as means ± SD and individual values, *n* = 14.**P < 0.01: differences from the Pre time point.

The P20 and P100 torques were significantly affected by the experimental exercise (Figure 2b, c). The P20 torque was significantly decreased postexercise (49.7% ± 17.8%, *P *< 0.001) compared with the pre‐exercise value (100%) and recovered only at 72 h after exercise (92.5% ± 15.0%, *P *> 0.05). As shown in Figure 2c, the P100 torque was decreased because of DJs (79.5% ± 15.4%, *P *< 0.01), but the P100 torque was restored at 12 h after exercise (91.9% ± 10.6%, *P *> 0.05). The decrease in the P20/P100 ratio showed that LFF exists after 50 DJs (61.3% ± 15.3%, *P *< 0.0001) (Figure 3). After 48 h of recovery, LFF was not fully recovered but was not significantly different from the pre‐exercise values (88.4% ± 13.0%, *P *> 0.05).

### 3.2 Delayed‐onset muscle soreness and CK activity

**FIGURE 4:**
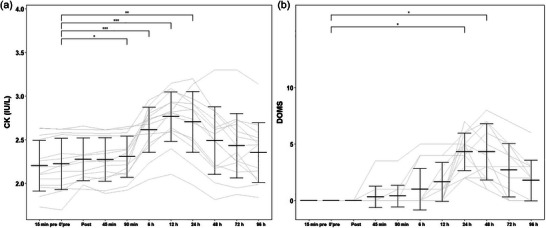
(a) CK. (b) DOMS (0−10 scale). Data are shown as mean ± SD and individual values, *n* = 14. *P < 0.05, **P < 0.01 and ***P < 0.001: differences from the Pre time point. Abbreviations: CK, creatine kinase; DOMS, delayed‐onset muscle soreness.

Plasma CK activity increased after DJ exercise (time effect: *P *< 0.001) (Figure 4a). A significant increase was found, from 2.22 ± 0.29 to 2.31 ± 0.24 IU/L on a log_10_ scale at 90 min postexercise (*P *< 0.05), to 2.62 ± 0.26 IU/L on a log_10_ scale at 6 h postexercise (*P *< 0.001), and CK values peaked at 12 h postexercise (2.77 ± 0.28 IU/L on a log_10_ scale, *P *< 0.001). A significant effect of time was found for muscle soreness (time effect: *P < *0.001), which peaked at 1−2 days postexercise (Figure 4b). At time points 24 and 48 h postexercise, all subjects reported having DOMS symptoms (4.31 ± 1.65 at 24 h postexercise, *P *< 0.05; 4.31 ± 2.50 at 48 h postexercise, *P *< 0.05). Muscle soreness (1−2 on a scale from 0 to 10) was reported by six participants at time point 96 h (1.77 ± 1.79), but it was not statistically significant compared with the pre‐exercise value (*P *> 0.05).

### 3.3 Lactate

We found a significant decrease in lactate concentration immediately postexercise (from 1.52 ± 0.29 to 1.21 ± 0.35 mmol/L, *P *= 0.016), and the decrease was still evident 45 min postexercise (1.2 ± 0.28 mmol/L, *P *= 0.012).

### 3.4 Plasma cfDNA concentration

**FIGURE 5:**
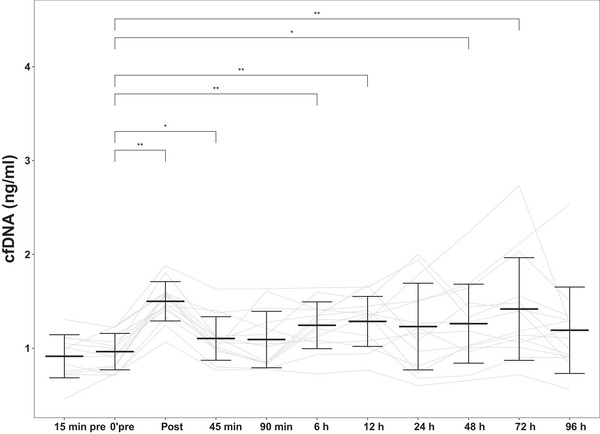
Cell‐free DNA (90 bp) kinetics over the assessment period. Data are shown as means ± SD on a logarithmic scale, *n* = 14. *P < 0.05 and **P < 0.01: differences from the Pre time point. Abbreviation: cfDNA, cell‐free DNA.

Cell‐free DNA (90 bp) values increased acutely after 50 DJs (from 0.97 ± 0.19 to 1.5 ± 0.21 ng/mL on a log_10_ scale, *P *< 0.01) and normalized 90 min postexercise (1.09 ± 0.3 ng/mL on a log_10_ scale, *P *> 0.05) (Figure 5). A significant increase in the cfDNA concentration was also noticed after 6 h (1.25 ± 0.25 ng/mL, *P *< 0.01), 12 h (1.29 ± 0.27 ng/mL, *P *< 0.01), 48 h (1.26 ± 0.41 ng/mL, *P *< 0.05) and 72 h (1.42 ± 0.55 ng/mL, *P *< 0.01). At 96 h after exercise, cfDNA values had recovered and did not differ significantly from pre‐exercise levels (*P* > 0.05).

### 3.5 Correlations

**FIGURE 6:**
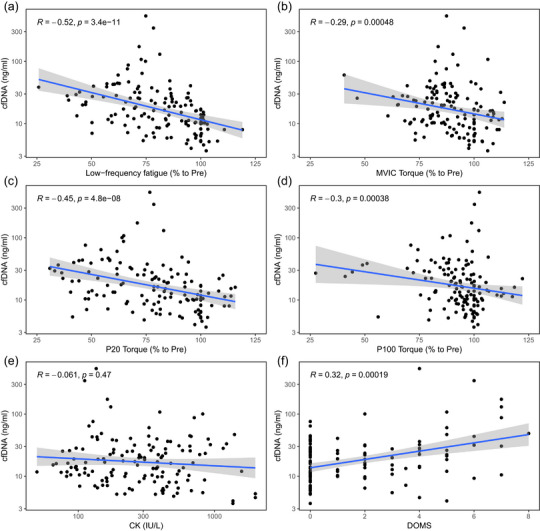
(a) Correlation between low‐frequency fatigue (ratio of 20 Hz to 100 Hz electrically stimulated torques) and log_10_‐transformed cfDNA, *n* = 14. (b) Correlation between MVIC torque and log_10_‐transformed cfDNA. (c) Correlation between P20 torque and log_10_‐transformed cfDNA, *n* = 14. (d) Correlation between P100 torque and log_10_‐transformed cfDNA, *n* = 14. (e) Correlation between log_10_‐transformed CK and log_10_‐transformed cfDNA, *n* = 14. (f) Correlation between DOMS and log_10_‐transformed cfDNA, *n* = 14. The lines represent the linear trend. The significance of the correlation and *r* values are displayed on the charts. Abbreviations: cfDNA, cell‐free DNA; CK, creatine kinase; DOMS, delayed‐onset muscle soreness; MVIC, maximal voluntary isometric contraction; P20, 1 s stimulation at 20 Hz; P100, 1 s stimulation at 100 Hz.

Cell‐free DNA levels were significantly correlated with LFF (*r *= −0.52, *P *= 3.4 × 10^−11^) (Figure 6a). The correlation was negative, indicating that greater increases in cfDNA were associated with a more pronounced LFF. Likewise, there was an association between cfDNA concentrations and P20 torque (*r *= −0.45, *P *= 4.8 × 10^−8^) (Figure 6c). Additionally, cfDNA levels were significantly correlated with P100 torque (*r *= −0.3, *P *= 0.00038) and MVIC torque (*r *= −0.29, *P *= 0.00048) (Figure 6b, d). We found no correlation with CK over all time points (*r *= −0.061, *P *= 0.47) (Figure 6e). However, there was a moderate correlation between cfDNA (90 bp) levels and DOMS (*r* = 0.32, *P *= 0.00019) (Figure 6f), where higher increases in cfDNA were associated with a greater degree of muscle soreness.

## 4 DISCUSSION

The effect of muscle‐damaging exercise without metabolic stress on cfDNA levels is unclear. Most studies have included exercises that are, for the most part, metabolically demanding (high metabolic stress), suggesting that this research is crucial in the context of cfDNA. Subjects performed repeated DJs at long (20 s) intervals, an exercise that has been shown to induce severe mechanical stress, whereas long intervals prevent any major metabolic challenges (Kamandulis et al., 2017). Furthermore, DJs are short intervals of work (∼1−2 s for a DJ), meaning that participants trained for a very short period (∼1−2 min). What has not been examined previously in any detail is the change in cfDNA kinetics after eccentric exercise without metabolic stress. Nor has the relationship been examined between cfDNA levels (postexercise or at 1, 2 or 3 days after exercise) and DOMS, CK and neuromuscular function (MVIC torque and LFF) following this type of exercise. Overall, the present study suggests that 50 DJs can lead to distinct time‐course changes in cfDNA levels. In comparison to other measured variables (CK, DOMS, MVIC and LFF) that were altered owing to either severe primary or secondary damage, only cfDNA changed in the presence of these two events as a result of 50 DJs. In addition, the magnitude of the increase in cfDNA was significantly correlated with LFF, MVIC and DOMS. Nevertheless, whether and how these changes depend on the DJ dosage must be explored.

Our study shows that 50 DJs performed at long intervals did not cause an accumulation of metabolites. Lactate did not increase after exercise, even though the exercise included repetitive eccentric contractions that induce mechanical stress. Other studies have shown that lactate levels decrease after exercise, suggesting accelerated lactate uptake by the muscles (Spengler et al., 1999). There, lactate is used as a substrate for energy production through oxidative phosphorylation and by enhanced lactate transport by monocarboxylate transporters on the surface of muscle cells (Gladden, 2004).

In the present study, the rise in plasma cfDNA occurred immediately postexercise and after 6, 12, 48 and 72 h, possibly reflecting primary damage (first increase) and local inflammation (second increase). Our results of moderate increases in cfDNA levels (∼5‐fold) were higher than the increases in cfDNA levels measured by Atamaniuk et al. (2010) after a single bout of high‐intensity strength exercise (six sets of six lifting exercises with 90%−95% of one‐repetition maximum) (3.3‐fold) but lower than the increases of cfDNA levels measured by Beiter et al. (2011) after exhaustive short‐term treadmill exercise (9.9‐fold). It has been reported that cfDNA levels depend on exercise modality, intensity and duration and are more pronounced after long‐lasting highly metabolically demanding exercise than after short low‐intensity exercise (Neuberger et al., 2022; Tug et al., 2017). The findings in this study show that only 1−2 min of mechanically demanding exercise, with no metabolic stress, can significantly increase cfDNA levels. In the present study, the initial elevation of cfDNA concentration might be linked to an aseptic inflammatory response (Tug et al., 2017) and caused by mechanical stress (Markus et al., 2021). If so, circulating cfDNA is released as an active secretion, and its levels immediately postexercise will be derived mainly from haematopoietic cells (Moss et al., 2018; Neuberger et al., 2022). An active release, which involves spontaneous release of cfDNA in free or encapsulated form, is the main mechanism of cfDNA release after acute exercise (Hu et al., 2021; Neuberger & Simon, 2022). Cell‐free DNA released by cell injury or death pathways, such as apoptosis or necrosis, refers to passive mechanisms (Grabuschnig et al., 2020; Han & Lo, 2021) and is usually a slow process. In the present study, the second increase in cfDNA levels at 6, 12, 48 and 72 h postexercise could be attributed to a local inflammatory reaction that happens through direct mechanical lesions of the muscle sarcomere (Brancaccio et al., 2010) and indirect formation of oxygen free radicals (Radak et al., 2008; Webb et al., 2017). Consequently, neutrophils infiltrate tissues owing to local inflammation and oxidative stress (Mortaz et al., 2018). Their role in releasing cfDNA is crucial (Moss et al., 2018; Neuberger et al., 2022) and thus might contribute to delayed elevation of the cfDNA level. It should be noted that we did not investigate the source of cells releasing cfDNA in this study. Further studies will be needed to identify the cells contributing to primary and secondary cfDNA release after muscle‐damaging exercise.

Structural damage to the contractile apparatus and cell membrane leads to reduced muscle function and local sterile inflammation (Paulsen et al., 2010, 2012). A decrease in muscle force‐generating capacity after eccentric exercise is a valuable tool as an indirect indicator of the severity of skeletal muscle damage. In the present study, MVIC was decreased immediately postexercise after 50 DJs. Muscle damage or fatigue can cause MVIC reductions soon after exercise (Souron et al., 2018). Given that CAR exhibited slight impairment throughout the assessment period, the decrease in MVIC torques following DJs might reflect muscular impairment rather than a decrease in neuronal activity (Kamandulis et al., 2022). Furthermore, LFF was significantly reduced immediately after exercise (∼40% decrease from Pre) and did not recover during the next 48 h postexercise. Similar to our results, Kamandulis et al. (2022) found an ∼45% reduction in LFF after 50 DJs. The underlying mechanisms of LFF differ depending on whether it is caused by metabolic or mechanical stress during exercise (Skurvydas et al., 2016). We recently reported a correlation between cfDNA levels and LFF during highly metabolically demanding exercise (Juškevičiūtė et al., 2023). The present study showed a stronger correlation between these two variables when only mechanical stress was applied, in comparison to our previous results on young subjects. Mechanically induced LFF is generally accepted to be caused by structural disturbances within muscle fibres (Kamandulis et al., 2017; Lauritzen et al., 2009), but impaired transmission between muscle fibres owing to distorted extracellular structural elements cannot be excluded (Kamandulis et al., 2022). A strong inflammatory response follows in response to these structural disturbances (Fatouros & Jamurtas, 2016), accompanied by an increase in neutrophil counts and other inflammatory cells, such as natural killer cells and lymphocytes (Stožer et al., 2020). Thus, neutrophil changes would contribute to increased cfDNA levels. Consequently, there is a correlation between elevated cfDNA levels and more pronounced LFF after exercise. Because the restoration of muscle function after exercise can be considered of clear practical value, the response of some additional variables, such as cfDNA, in the days following exercise should also be monitored for signs of improvement.

In addition, we wanted to determine whether the postexercise cfDNA concentration could be associated with secondary muscle damage markers, such as CK and DOMS. However, there was no significant correlation between the magnitude of the increase in plasma cfDNA concentration and CK. The elevation of CK concentration, which did not return to baseline values by the end of the experiment, in our study was similar to that observed by Kamandulis et al. (2022) following 50 DJs in healthy young male subjects. Studies show that there is often a poor relationship between functional outcomes and CK activity (Koch et al., 2014), as evidenced by the results of our study (no correlation was found between CK and LFF, *r* = −0.04, *P *= 0.64). In line with our results, Andreatta et al. (2018) showed a better correlation of cfDNA with muscle performance (they evaluated muscular performance by both squat jump and countermovement tests) than CK concentration after leg‐press exercise with high and low intensity. However, in contrast to our findings, significantly increased lactate levels were found in their study (Andreatta et al., 2018).

Delayed muscle soreness is associated with the inflammatory response and muscle damage (Cheung et al., 2003), and we hypothesized that postexercise DOMS might be linked to elevated cfDNA levels. We found a significant correlation between cfDNA and DOMS. Studies have found several mechanisms associated with DOMS, including mechanical strains in muscle structures, overstretching sarcomeres, biochemical reactions that affect muscle contractile structures, and inflammatory processes related to oxidative stress and heightened sensitivity of pain receptors (da Silva et al., 2021). In previous studies, cfDNA has been associated with increased pain perception (Fleckenstein et al., 2021). There is also a link between cfDNA and pain in conditions such as sickle‐cell disease (Al‐humood et al., 2014) and *Bothrops* envenomation (de Souza Barbosa et al., 2021). As a physiological phenomenon, soreness occurs around extracellular structures, the fascia of the muscles and the nervous system (sensitization) (Paulsen & Benestad, 2019). More precisely, sensations of muscle soreness could result from a complex interaction of damage to the muscle structure, disrupted Ca^2+^ homeostasis or sensitization of nociceptors from inflammatory cell infiltrates (Hyldahl & Hubal, 2014). Sensitization of the peripheral and central nervous systems, including activation of nociceptors, is linked to the immune system and neuroinflammation (Starobova et al., 2020). In the early stages of inflammation, neutrophils, macrophages and mast cells can be recruited to relevant tissues, thereby initiating the maintenance and resolution of pain (Yang et al., 2022). Activation of an immune response modulates the excitability of pain pathways by forming an integrated network of immune cells, glia and neurons (Fleckenstein et al., 2021). Enhanced activation of immune cells, during and after exercise, might result in a more pronounced correlation between cfDNA and DOMS.

This study had limitations. Only young healthy men were included in this study, hence our results cannot be extrapolated to the general population, including females, older people and people with medical conditions. Moreover, there is controversy regarding sex‐based differences in circulating cfDNA levels (Yuwono et al., 2021). Future studies should be expanded to include individuals of both sexes and all ages (Jylhava et al., 2011; Zhong et al., 2007).

## AUTHOR CONTRIBUTIONS

Marius Brazaitis, Nerijus Eimantas and Ema Juškevičiūtė were responsible for the conception or design of the work and conduction of the experiments. Ema Juškevičiūtė, Marius Brazaitis, Kirsten Heinkel, Elmo Neuberger and Perikles Simon were responsible for analyzing and interpreting the data. All authors critically revised the work for important intellectual content. All authors have read and approved the manuscript and agree to be acountable for all aspects of the work in ensuring that questions related to the accuracy or integrity of any part of the work are appropriately investigated and resolved. All persons designated as authors qualify for authorship, and all those who qualify for authorship are listed.

## CONFLICT OF INTEREST

None.

## FUNDING INFORMATION

None.

## Data Availability

The data that support the findings of the present study are available from the corresponding author on reasonable request.

## References

[eph13582-bib-0001] Al‐Humood, S. , Zueriq, R. , Al‐Faris, L. , Marouf, R. , & Al‐Mulla, F. (2014). Circulating cell‐free DNA in sickle cell disease: Is it a potentially useful biomarker? Archives of Pathology and Laboratory Medicine, 138(5), 678–683. 24786126 10.5858/arpa.2012-0725-OA

[eph13582-bib-0002] Andreatta, M. V. , Curty, V. M. , Coutinho, J. V. S. , MÂA, S. , Vassallo, P. F. , de Sousa, N. F. , & Barauna, V. G. (2018). Cell‐free DNA as an earlier predictor of exercise‐induced performance decrement related to muscle damage. International Journal of Sports Physiology and Performance, 13(7), 953–956. 29182414 10.1123/ijspp.2017-0421

[eph13582-bib-0003] Atamaniuk, J. , Stuhlmeier, K. M. , Vidotto, C. , Tschan, H. , Dossenbach‐Glaninger, A. , & Mueller, M. M. (2008). Effects of ultra‐marathon on circulating DNA and mRNA expression of pro‐and anti‐apoptotic genes in mononuclear cells. European Journal of Applied Physiology, 104, 711–717. 18651163 10.1007/s00421-008-0827-2

[eph13582-bib-0004] Atamaniuk, J. , Vidotto, C. , Kinzlbauer, M. , Bachl, N. , Tiran, B. , & Tschan, H. (2010). Cell‐free plasma DNA and purine nucleotide degradation markers following weightlifting exercise. European Journal of Applied Physiology, 110, 695–701. 20577758 10.1007/s00421-010-1532-5

[eph13582-bib-0005] Beck, C. R. , Collier, P. , Macfarlane, C. , Malig, M. , Kidd, J. M. , Eichler, E. E. , Badge, R. M. , & Moran, J. V. (2010). LINE‐1 retrotransposition activity in human genomes. Cell, 141(7), 1159–1170. 20602998 10.1016/j.cell.2010.05.021 PMC3013285

[eph13582-bib-0006] Beiter, T. , Fragasso, A. , Hudemann, J. , Nieß, A. M. , & Simon, P. (2011). Short‐term treadmill running as a model for studying cell‐free DNA kinetics in vivo. Clinical Chemistry, 57(4), 633–636. 21296972 10.1373/clinchem.2010.158030

[eph13582-bib-0007] Brancaccio, P. , Lippi, G. , & Maffulli, N. (2010). Biochemical markers of muscular damage. Clinical Chemistry and Laboratory Medicine, 48(6), 757–767. 20518645 10.1515/CCLM.2010.179

[eph13582-bib-0008] Breitbach, S. , Tug, S. , & Simon, P. (2012). Circulating cell‐free DNA: An up‐coming molecular marker in exercise physiology. Sports Medicine, 42, 565–586. 22694348 10.2165/11631380-000000000-00000

[eph13582-bib-0009] Chalchat, E. , Gaston, A. F. , Charlot, K. , Peñailillo, L. , Valdés, O. , Tardo‐Dino, P. E. , Nosaka, K. , Martin, V. , Garcia‐Vicencio, S. , & Siracusa, J. (2022). Appropriateness of indirect markers of muscle damage following lower limbs eccentric‐biased exercises: A systematic review with meta‐analysis. PLoS ONE, 17(7), e0271233. 35834532 10.1371/journal.pone.0271233 PMC9282447

[eph13582-bib-0010] Cheung, K. , Hume, P. A. , & Maxwell, L. (2003). Delayed onset muscle soreness: Treatment strategies and performance factors. Sports Medicine, 33, 145–164. 12617692 10.2165/00007256-200333020-00005

[eph13582-bib-0011] Clarkson, P. M. , & Hubal, M. J. (2002). Exercise‐induced muscle damage in humans. American Journal of Physical Medicine & Rehabilitation, 81(11), S52–S69. 12409811 10.1097/00002060-200211001-00007

[eph13582-bib-0012] Damas, F. , Nosaka, K. , Libardi, C. A. , Chen, T. C. , & Ugrinowitsch, C. (2016). Susceptibility to exercise‐induced muscle damage: A cluster analysis with a large sample. International Journal of Sports Medicine, 37(8), 633–640. 27116346 10.1055/s-0042-100281

[eph13582-bib-0013] Dargeviciute, G. , Masiulis, N. , Kamandulis, S. , Skurvydas, A. , & Westerblad, H. (2013). Residual force depression following muscle shortening is exaggerated by prior eccentric drop jump exercise. Journal of Applied Physiology, 115(8), 1191–1195. 23928115 10.1152/japplphysiol.00686.2013

[eph13582-bib-0014] da Silva, W. , Machado, Á. S. , Lemos, A. L. , de Andrade, C. F. , Priego‐Quesada, J. I. , & Carpes, F. P. (2021). Relationship between exercise‐induced muscle soreness, pain thresholds, and skin temperature in men and women. Journal of Thermal Biology, 100, 103051. 34503798 10.1016/j.jtherbio.2021.103051

[eph13582-bib-0015] Dias, S. S. , Weber, M. G. , Padoin, S. , Andrello, A. C. , Jussiani, E. I. , de, P. , & Ramos, S. (2022). Circulating concentration of chemical elements during exercise‐induced muscle damage and the repeated Bout effect. Biological Trace Element Research, 200(3), 1060–1070. 33904125 10.1007/s12011-021-02737-8

[eph13582-bib-0016] Dupuy, O. , Douzi, W. , Theurot, D. , Bosquet, L. , & Dugué, B. (2018). An evidence‐based approach for choosing post‐exercise recovery techniques to reduce markers of muscle damage, soreness, fatigue, and inflammation: A systematic review with meta‐analysis. Frontiers in Physiology, 9, 403. 29755363 10.3389/fphys.2018.00403 PMC5932411

[eph13582-bib-0017] Eimantas, N. , Ivanove, S. , Baranauskiene, N. , Solianik, R. , & Brazaitis, M. (2022). Modulation of neuromuscular excitability in response to acute noxious heat exposure has no additional effects on central and peripheral fatigability. Frontiers in Physiology, 13, 936885. 36035478 10.3389/fphys.2022.936885 PMC9412021

[eph13582-bib-0018] Fatouros, I. G. , & Jamurtas, A. Z. (2016). Insights into the molecular etiology of exercise‐induced inflammation: Opportunities for optimizing performance. Journal of Inflammation Research, 9, 175–186. 27799809 10.2147/JIR.S114635 PMC5085309

[eph13582-bib-0019] Fleckenstein, J. , Neuberger, E. W. , Bormuth, P. , Comes, F. , & Schneider, A. (2021). Investigation of the sympathetic regulation in delayed onset muscle soreness: Results of an RCT. Frontiers in Physiology, 12, 697335. 34603072 10.3389/fphys.2021.697335 PMC8481669

[eph13582-bib-0020] Friden, J. , & Lieber, R. L. (2001). Eccentric exercise‐induced injuries to contractile and cytoskeletal muscle fibre components. Acta Physiologica Scandinavica, 171(3), 321–326. 11412144 10.1046/j.1365-201x.2001.00834.x

[eph13582-bib-0021] Gladden, L. B. (2004). Lactate metabolism during exercise. Medicine and Sport Science, 46, 152–196.

[eph13582-bib-0022] Grabuschnig, S. , Bronkhorst, A. J. , Holdenrieder, S. , Rodriguez, I. R. , Schliep, K. P. , Schwendenwein, D. , Ungerer, V. , & Sensen, C. W. (2020). Putative origins of cell‐free DNA in humans: A review of active and passive nucleic acid release mechanisms. International Journal of Molecular Sciences, 21(21), 1–24. 10.3390/ijms21218062 PMC7662960 33137955

[eph13582-bib-0023] Haller, N. , Behringer, M. , Reichel, T. , Wahl, P. , Simon, P. , Krüger, K. , Zimmer, P. , & Stöggl, T. (2023). Blood‐based biomarkers for managing workload in athletes: Considerations and recommendations for evidence‐based use of established biomarkers. Sports Medicine, 53(7), 1315–1333. 37204619 10.1007/s40279-023-01836-x PMC10197055

[eph13582-bib-0024] Haller, N. , Tug, S. , Breitbach, S. , Jörgensen, A. , & Simon, P. (2017). Increases in circulating cell‐free DNA during aerobic running depend on intensity and duration. International Journal of Sports Physiology and Performance, 12(4), 455–462. 27617389 10.1123/ijspp.2015-0540

[eph13582-bib-0025] Han, D. S. , & Lo, Y. D. (2021). The nexus of cfDNA and nuclease biology. Trends in Genetics, 37(8), 758–770. 34006390 10.1016/j.tig.2021.04.005

[eph13582-bib-0026] Hu, Z. , Chen, H. , Long, Y. , Li, P. , & Gu, Y. (2021). The main sources of circulating cell‐free DNA: Apoptosis, necrosis and active secretion. Critical Reviews in Oncology/Hematology, 157, 103166. 33254039 10.1016/j.critrevonc.2020.103166

[eph13582-bib-0027] Humińska‐Lisowska, K. , Mieszkowski, J. , Kochanowicz, A. , Stankiewicz, B. , Niespodziński, B. , Brzezińska, P. , Ficek, K. , Kemerytė‐Ivanauskienė, E. , & Cięszczyk, P. (2021). cfDNA changes in maximal exercises as a sport adaptation predictor. Genes, 12(8), 1238. 34440412 10.3390/genes12081238 PMC8392318

[eph13582-bib-0028] Hyldahl, R. D. , & Hubal, M. J. (2014). Lengthening our perspective: Morphological, cellular, and molecular responses to eccentric exercise. Muscle & Nerve, 49(2), 155–170. 24030935 10.1002/mus.24077

[eph13582-bib-0029] Juškevičiūtė, E. , Neuberger, E. , Eimantas, N. , Venckunas, T. , Kamandulis, S. , Simon, P. , & Brazaitis, M. (2023). Three‐week sprint interval training (SIT) reduces cell‐free DNA and low‐frequency fatigue but does not induce VO2max improvement in older men. European Journal of Applied Physiology, 124(4), 1297–1309. 38015284 10.1007/s00421-023-05366-2

[eph13582-bib-0030] Jylhävä, J. , Kotipelto, T. , Raitala, A. , Jylhä, M. , Hervonen, A. , & Hurme, M. (2011). Aging is associated with quantitative and qualitative changes in circulating cell‐free DNA: The Vitality 90+ study. Mechanisms of Ageing and Development, 132(1–2), 20–26. 21078336 10.1016/j.mad.2010.11.001

[eph13582-bib-0031] Kamandulis, S. , De Souza Leite, F. , Hernández, A. , Katz, A. , Brazaitis, M. , Bruton, J. D. , Venckunas, T. , Masiulis, N. , Mickeviciene, D. , Eimantas, N. , Subocius, A. , Rassier Di, E. , Skurvydas, A. , Ivarsson, N. , & Westerblad, H. (2017). Prolonged force depression after mechanically demanding contractions is largely independent of Ca^2+^ and reactive oxygen species. Federation of American Societies of Experimental Biology Journal, 31(11), 4809–4820. 10.1096/fj.201700019R 28716970

[eph13582-bib-0032] Kamandulis, S. , Mickevicius, M. , Snieckus, A. , Streckis, V. , Montiel‐Rojas, D. , Chaillou, T. , Westerblad, H. , & Venckunas, T. (2022). Increasing the resting time between drop jumps lessens delayed‐onset muscle soreness and limits the extent of prolonged low‐frequency force depression in human knee extensor muscles. European Journal of Applied Physiology, 122, 255–266. 34674024 10.1007/s00421-021-04834-x

[eph13582-bib-0033] Koch, A. J. , Pereira, R. , & Machado, M. (2014). The creatine kinase response to resistance exercise. Journal of Musculoskeletal & Neuronal Interactions, 14(1), 68–77. 24583542

[eph13582-bib-0034] Korabecna, M. , Zinkova, A. , Brynychova, I. , Chylikova, B. , Prikryl, P. , Sedova, L. , Neuzil, P. , & Seda, O. (2020). Cell‐free DNA in plasma as an essential immune system regulator. Scientific Reports, 10(1), 17478. 33060738 10.1038/s41598-020-74288-2 PMC7566599

[eph13582-bib-0035] Lau, W. Y. , Blazevich, A. J. , Newton, M. J. , Wu, S. S. X. , & Nosaka, K. (2015). Assessment of muscle pain induced by elbow‐flexor eccentric exercise. Journal of Athletic Training, 50(11), 1140–1148. 26523661 10.4085/1062-6050-50.11.05 PMC4732393

[eph13582-bib-0036] Lauritzen, F. , Paulsen, G. , Raastad, T. , Bergersen, L. H. , & Owe, S. G. (2009). Gross ultrastructural changes and necrotic fiber segments in elbow flexor muscles after maximal voluntary eccentric action in humans. Journal of Applied Physiology, 107(6), 1923–1934. 19797695 10.1152/japplphysiol.00148.2009

[eph13582-bib-0037] Markus, I. , Constantini, K. , Hoffman, J. R. , Bartolomei, S. , & Gepner, Y. (2021). Exercise‐induced muscle damage: Mechanism, assessment and nutritional factors to accelerate recovery. European Journal of Applied Physiology, 121, 969–992. 33420603 10.1007/s00421-020-04566-4

[eph13582-bib-0038] Mavropalias, G. , Calapre, L. , Morici, M. , Koeda, T. , Poon, W. C. K. , Barley, O. R. , Gray, E. , Blazevich, A. J. , & Nosaka, K. (2021). Changes in plasma hydroxyproline and plasma cell‐free DNA concentrations after higher‐ versus lower‐intensity eccentric cycling. European Journal of Applied Physiology, 121(4), 1087–1097. 33439308 10.1007/s00421-020-04593-1

[eph13582-bib-0039] Mortaz, E. , Alipoor, S. D. , Adcock, I. M. , Mumby, S. , & Koenderman, L. (2018). Update on neutrophil function in severe inflammation. Frontiers in Immunology, 9, 411381. 10.3389/fimmu.2018.02171 PMC6190891 30356867

[eph13582-bib-0040] Moss, J. , Magenheim, J. , Neiman, D. , Zemmour, H. , Loyfer, N. , Korach, A. , Samet, Y. , Maoz, M. , Druid, H. , Arner, P. , Fu, K. Y. , Kiss, E. , Spalding, K. L. , Landesberg, G. , Zick, A. , Grinshpun, A. , Shapiro, A. M. J. , Grompe, M. , Wittenberg, A. D. , … Dor, Y. (2018). Comprehensive human cell‐type methylation atlas reveals origins of circulating cell‐free DNA in health and disease. Nature Communications, 9(1), 5068. 10.1038/s41467-018-07466-6 PMC6265251 30498206

[eph13582-bib-0041] Muanjai, P. , Mickevičius, M. , Sniečkus, A. , Satkunskiene, D. , Kamandulis, S. , & Jones, D. A. (2019). Slow torque recovery after eccentric exercise and the repeated bout effect; the role of primary and secondary muscle damage. Journal of Musculoskeletal & Neuronal Interactions, 19(2), 207–214. 31186391 PMC6587094

[eph13582-bib-0042] Neuberger, E. W. , & Simon, P. (2022). Cell‐free DNA in sports medicine: Implications for clinical laboratory medicine. Journal of Laboratory Medicine, 46(4), 295–300.

[eph13582-bib-0043] Neuberger, E. W. , Sontag, S. , Brahmer, A. , Philippi, K. F. , Radsak, M. P. , Wagner, W. , & Simon, P. (2022). Physical activity specifically evokes release of cell‐free DNA from granulocytes thereby affecting liquid biopsy. Clinical Epigenetics, 14(1), 29. 35193681 10.1186/s13148-022-01245-3 PMC8864902

[eph13582-bib-0044] Newham, D. J. , Jones, D. A. , & Clarkson, P. M. (1987). Repeated high‐force eccentric exercise: Effects on muscle pain and damage. Journal of Applied Physiology, 63(4), 1381–1386. 3693172 10.1152/jappl.1987.63.4.1381

[eph13582-bib-0045] Paulsen, G. , & Benestad, H. B. (2019). Muscle soreness and rhabdomyolysis. Tidsskrift for Den norske legeforening: tidsskrift for praktisk medicin, ny raekke, 139(10). 10.4045/tidsskr.18.0727 31238673

[eph13582-bib-0046] Paulsen, G. , Crameri, R. , Benestad, H. B. , Fjeld, J. G. , Mørkrid, L. , Hallén, J. , & Raastad, T. (2010). Time course of leukocyte accumulation in human muscle after eccentric exercise. Medicine & Science in Sports & Exercise, 42(1), 75–85. 20010127 10.1249/MSS.0b013e3181ac7adb

[eph13582-bib-0047] Paulsen, G. , Ramer Mikkelsen, U. , Raastad, T. , & Peake, J. M. (2012). Leucocytes, cytokines and satellite cells: What role do they play in muscle damage and regeneration following eccentric exercise? Exercise Immunology Review, 18, 42–97. 22876722

[eph13582-bib-0048] Peake, J. M. , Neubauer, X. O. , Della Gatta, P. A. , & Nosaka, X. K. (2017). Recovery from Exercise Muscle damage and inflammation during recovery from exercise. Journal of Applied Physiology, 122, 559–570. 28035017 10.1152/japplphysiol.00971.2016

[eph13582-bib-0049] Radak, Z. , Chung, H. Y. , Koltai, E. , Taylor, A. W. , & Goto, S. (2008). Exercise, oxidative stress and hormesis. Ageing Research Reviews, 7(1), 34–42. 17869589 10.1016/j.arr.2007.04.004

[eph13582-bib-0050] Saw, A. E. , Main, L. C. , & Gastin, P. B. (2016). Monitoring the athlete training response: Subjective self‐reported measures trump commonly used objective measures: A systematic review. British Journal of Sports Medicine, 50(5), 281–291. BMJ Publishing Group. 26423706 10.1136/bjsports-2015-094758 PMC4789708

[eph13582-bib-0051] Skurvydas, A. , Brazaitis, M. , Venckūnas, T. , & Kamandulis, S. (2011). Predictive value of strength loss as an indicator of muscle damage across multiple drop jumps. Applied Physiology, Nutrition, and Metabolism, 36(3), 353–360. 10.1139/h11-023 21574783

[eph13582-bib-0052] Skurvydas, A. , Mamkus, G. , Kamandulis, S. , Dudoniene, V. , Valanciene, D. , & Westerblad, H. (2016). Mechanisms of force depression caused by different types of physical exercise studied by direct electrical stimulation of human quadriceps muscle. European Journal of Applied Physiology, 116, 2215–2224. 27637589 10.1007/s00421-016-3473-0 PMC5118408

[eph13582-bib-0053] Souron, R. , Nosaka, K. , & Jubeau, M. (2018). Changes in central and peripheral neuromuscular fatigue indices after concentric versus eccentric contractions of the knee extensors. European Journal of Applied Physiology, 118, 805–816. 29411127 10.1007/s00421-018-3816-0

[eph13582-bib-0054] de Souza Barbosa, Ê. , Ibiapina, H. N. S. , da Silva, S. R. , Costa, A. G. , Val, F. F. , & Mendonça‐da‐Silva, I. (2021). Association of cfDNA levels and bothrops envenomation. Toxicon, 192, 66–73. 33497746 10.1016/j.toxicon.2021.01.015

[eph13582-bib-0055] Spengler, C. M. , Roos, M. , Laube, S. M. , & Boutellier, U. (1999). Decreased exercise blood lactate concentrations after respiratory endurance training in humans. European Journal of Applied Physiology and Occupational Physiology, 79, 299–305. 10090627 10.1007/s004210050511

[eph13582-bib-0056] Starobova, H. , Nadar, E. I. , & Vetter, I. (2020). The NLRP3 Inflammasome: Role and Therapeutic Potential in Pain Treatment. Frontiers in Physiology, 11, 543422. Frontiers Media S.A. 10.3389/fphys.2020.01016 PMC7468416 32973552

[eph13582-bib-0057] Stožer, A. , Vodopivc, P. , & Bombek, L. K. (2020). Pathophysiology of exercise‐induced muscle damage and its structural, functional, metabolic, and clinical consequences. Physiological Research, 69(4), 565. 32672048 10.33549/physiolres.934371 PMC8549894

[eph13582-bib-0058] Tanabe, Y. , Fujii, N. , & Suzuki, K. (2022). Dietary supplementation for attenuating exercise‐induced muscle damage and delayed‐onset muscle soreness in humans. In Nutrients, 14(1), 70. MDPI. 10.3390/nu14010070 PMC8746365 35010943

[eph13582-bib-0059] Treigyte, V. , Chaillou, T. , Eimantas, N. , Venckunas, T. , & Brazaitis, M. (2024). Passive heating‐induced changes in muscle contractile function are not further augmented by prolonged exposure in young males experiencing moderate thermal stress. Frontiers in Physiology, 15, 1356488. 38476145 10.3389/fphys.2024.1356488 PMC10928533

[eph13582-bib-0060] Tug, S. , Tross, A. K. , Hegen, P. , Neuberger, E. W. I. , Helmig, S. , Schöllhorn, W. , & Simon, P. (2017). Acute effects of strength exercises and effects of regular strength training on cell free DNA concentrations in blood plasma. PLoS ONE, 12(9), e0184668. 28910365 10.1371/journal.pone.0184668 PMC5599009

[eph13582-bib-0061] Webb, R. , Hughes, M. G. , Thomas, A. W. , & Morris, K. (2017). The ability of exercise‐associated oxidative stress to trigger redox‐sensitive signalling responses. Antioxidants, 6(3), 63. 28796154 10.3390/antiox6030063 PMC5618091

[eph13582-bib-0062] Wyckelsma, V. L. , Venckunas, T. , Brazaitis, M. , Gastaldello, S. , Snieckus, A. , Eimantas, N. , Baranauskiene, N. , Subocius, A. , Skurvydas, A. , Pääsuke, M. , Gapeyeva, H. , Kaasik, P. , Pääsuke, R. , Jürimäe, J. , Graf, B. A. , Kayser, B. , Place, N. , Andersson, D. C. , Kamandulis, S. , & Westerblad, H. (2020). Vitamin c and e treatment blunts sprint interval training–induced changes in inflammatory mediator‐, calcium‐, and mitochondria‐related signaling in recreationally active elderly humans. Antioxidants, 9(9), 1–20. 10.3390/antiox9090879 PMC7555371 32957522

[eph13582-bib-0063] Yamada, R. , Himori, K. , Tatebayashi, D. , Ashida, Y. , Ikezaki, K. , Miyata, H. , Kanzaki, K. , Wada, M. , Westerblad, H. , & Yamada, T. (2018). Preconditioning contractions prevent the delayed onset of myofibrillar dysfunction after damaging eccentric contractions. The Journal of Physiology, 596(18), 4427–4442. 30062729 10.1113/JP276026 PMC6138287

[eph13582-bib-0064] Yang, J. X. , Wang, H. F. , Chen, J. Z. , Li, H. Y. , & Hu, J. C. (2022). Potential neuroimmune interaction in chronic pain: A review on immune cells in peripheral and central sensitization. Frontiers in Pain Research, 3, 946846. 35859655 10.3389/fpain.2022.946846 PMC9289261

[eph13582-bib-0065] Yuwono, N. L. , Warton, K. , & Ford, C. E. (2021). The influence of biological and lifestyle factors on circulating cell‐free DNA in blood plasma. eLife, 10, e69679. 34752217 10.7554/eLife.69679 PMC8577835

[eph13582-bib-0066] Zhong, X. Y. , Hahn, S. , Kiefer, V. , & Holzgreve, W. (2007). Is the quantity of circulatory cell‐free DNA in human plasma and serum samples associated with gender, age and frequency of blood donations? Annals of Hematology, 86, 139–143. 17024502 10.1007/s00277-006-0182-5

